# Extracellular vesicles from young women’s breast cancer patients drive increased invasion of non-malignant cells via the Focal Adhesion Kinase pathway: a proteomic approach

**DOI:** 10.1186/s13058-020-01363-x

**Published:** 2020-11-23

**Authors:** Kimberly R. Jordan, Jessica K. Hall, Troy Schedin, Michelle Borakove, Jenny J. Xian, Monika Dzieciatkowska, Traci R. Lyons, Pepper Schedin, Kirk C. Hansen, Virginia F. Borges

**Affiliations:** 1 Young Women’s Breast Cancer Translational Program, Division of Medical Oncology, Department of Medicine, University of Colorado Anschutz Medical Campus, Aurora, CO USA; 2 Department of Immunology and Microbiology, School of Medicine, University of Colorado Anschutz Medical Campus, Aurora, CO USA; 3 School of Medicine, University of Maryland, Baltimore, MD USA; 4 Department of Biochemistry and Molecular Genetics, School of Medicine, University of Colorado Anschutz Medical Campus, Aurora, CO USA; 5 Knight Cancer Institute and Department of Cell, Developmental & Cancer Biology, Oregon Health Science University, Portland, OR USA

**Keywords:** Breast cancer, Young women’s breast cancer, Extracellular vesicles, Nanoparticles, Exosomes, Proteomics

## Abstract

**Background:**

Extracellular vesicles (EVs) are small membrane particles that contribute to cancer progression and metastases by transporting biologically significant proteins and nucleic acids. They may also serve as biomarkers of various disease states or important therapeutic targets. Breast cancer EVs have the potential to change the behavior of other cells in their microenvironment. However, the proteomic content of EVs isolated from young women’s breast cancer patients and the mechanisms underlying the influence of EVs on tumor cell behavior have not yet been reported.

**Methods:**

In our current translational studies, we compared the proteomic content of EVs isolated from invasive breast cancer cell lines and plasma samples from young women’s breast cancer (YWBC) patients and age-matched healthy donors using mass spectrometry. We analyzed the functionality of EVs in two dimensional tumor cell invasion assays and the gene expression changes in tumor cells after incubation with EVs.

**Results:**

We found that treatment with EVs from both invasive breast cancer cell lines and plasma of YWBC patients altered the invasive properties of non-invasive breast cancer cells. Proteomics identified differences between EVs from YWBC patients and healthy donors that correlated with their altered function. Further, we identified gene expression changes in non-invasive breast cancer cells after treatment with EVs that implicate the Focal Adhesion Kinase (FAK) signaling pathway as a potential targetable pathway affected by breast cancer-derived EVs.

**Conclusions:**

Our results suggest that the proteome of EVs from breast cancer patients reflects their functionality in tumor motility assays and may help elucidate the role of EVs in breast cancer progression.

## Background

Despite recent advances in targeted therapy for specific breast cancer subtypes, breast cancer continues to cause ~ 40,000 deaths in the USA annually, remains the second leading type of women’s cancer, and is the leading cancer diagnosis in young, premenopausal women [1]. Nearly 27,000 American women under the age of 45 are diagnosed with young women’s breast cancer (YWBC) each year. Compared to women diagnosed over the age of 45, patients with YWBC have a poorer prognosis, increased metastases, and an increased risk of death [1–3]. The causes of increased metastases in YWBC are being actively explored with the goal of identifying targetable treatments for this high-risk age group. Our Young Women’s Breast Cancer Translational Program focuses specifically on this risk group and identifying unique aspects of a young onset breast cancer that may be exploitable as prognostic or predictive biomarkers or therapeutic targets. While our research focuses specifically on YWBC, these findings may also be applicable to breast cancer patients of all age groups.

Extracellular vesicles (EVs) are cell-derived nanoparticles with a characteristic double membrane that contain nucleic acids and proteins, including microRNA, mRNA, non-coding RNA, DNA, transcription factors, integrins, signaling molecules, and growth factors [4, 5]. Although EVs were discovered in the late 1970s, their importance in disease states such as cancer and inflammation has only recently been appreciated by the wider scientific community [6–10]. EVs enable local communication between neighboring cells and cells in distant locations by traveling through various biologic fluids such as blood, urine, and saliva [11–14]. In cancer, EVs have been shown to increase tumor growth, to enhance tumor cell invasion, and to potentially establish permissive microenvironments that enable tumor cell metastasis [14–17]. Important for cancer patient diagnosis and prognosis, EVs hold promise as a diagnostic and/or monitoring tool of a patient’s disease state and may provide biomarkers for patient outcomes and/or responses to cancer treatments [18–23]. Furthermore, EVs are emerging as an important tool in drug delivery and vaccine design and may be targets of future cancer therapies [24–29]. More research is needed to understand the importance of breast cancer-derived EVs in human disease and their potential as diagnostics or therapeutic targets.

Several studies have demonstrated that breast cancer cells secrete EVs containing functional molecules with the potential to change the behavior of other cells in their microenvironment [28, 30, 31]. One group has reported the proteomic content of breast cancer patient EVs in largely postmenopausal women [32]. However, the content of EVs isolated from young women’s breast cancer patients and the proteomic mechanisms underlying the influence of EVs on tumor cell behavior have not yet been reported. Here, we demonstrate the distinct proteomic content of EVs from invasive breast cancer cell lines compared to non-invasive breast cancer cells. These proteomic differences may account for the ability of tumor-derived EVs to induce cell invasion. Similarly, we compare the invasive effects of EVs isolated from the peripheral blood of YWBC patients and healthy donors and identify proteins that may contribute to the increased invasive effects of EVs from YWBC patients. Furthermore, we identify downstream signaling pathways, including the Focal Adhesion Kinase (FAK) pathway, that are altered in non-invasive breast cancer cells after co-incubation with EVs from invasive breast cancer cells and from YWBC patients, which may serve as targets for intervention.

FAK is a cytoplasmic non-receptor protein kinase that drives cancer cell proliferation, survival, invasion, and epithelial-to-mesenchymal transition (EMT) [33, 34]. FAK mRNA is increased in invasive breast cancers and ovarian tumors and correlates with poor overall survival [33, 35–37]. Previous studies have reported elevated levels of FAK in cancer-derived EVs, including breast cancer; however, a functional link between FAK signaling and the phenotypic effects of breast cancer EVs has not previously been demonstrated [33, 38–40]. We find that the Focal Adhesion Kinase (FAK) pathway is affected in breast cancer cells treated with EVs and show that inhibition of the FAK pathway may mitigate the invasive effects of breast cancer EVs.

## Materials and methods

### Human plasma collection

Whole blood was collected in sodium citrate tubes under Colorado Multiple Institutional Review Board (COMIRB) approved protocol. YWBC patients were between the ages of 18 and 45 and had no known autoimmune condition, no other significant comorbid conditions (i.e., active infection, heart disease, diabetes), no other diagnosis of other concurrent disease, and no systemic drug treatment or surgery prior to blood draw (see Additional file 1 for clinical details). Proteomic analysis of EVs included 10 nulliparous patients and 10 parous patients (1–6 children, time range since last pregnancy 0.33–4 years). Invasion assay analysis of EVs included 8 nulliparous patients and 10 parous patients (1–6 children, time range since last pregnancy 0.33–4 years). Age-matched healthy female donors had never been diagnosed with cancer, an autoimmune disorder, or any of the comorbid conditions listed above and had reported never having been pregnant. Nulliparous healthy donors were chosen as controls for this study because prior pregnancy is a known risk factor for YWBC and the role of EVs during and after pregnancy on subsequent breast cancer risk is unknown [41, 42]. Study data were collected and managed using REDCap electronic data capture tools hosted at the University of Colorado Anschutz Medical Campus [43]. Plasma was separated by centrifugation at 2000×*g* for 15 min at room temperature. The supernatant was collected and centrifuged at 2000×*g* for an additional 10 min at room temperature and stored at − 80 °C.

### EV isolation

Plasma samples were thawed on ice and spun at 15,000×*g* for 10 min at room temperature. One milliliter of supernatant was collected and layered over a 1.5 × 10 cm high Sepharose CL-2B size-exclusion column (GE Healthcare, UK). Thirty 1-ml serial fractions were eluted by gravity filtration with 0.32% sodium citrate in PBS as previously described for EV isolation [44]. Fractions were analyzed for the presence of EVs by nanoparticle tracking analysis. Fractions 5 through 10 were identified as enriched in EVs and combined and concentrated using 100-kDa molecular weight cutoff ultrafiltration tubes (Sartorius). These purified EVs were either stored at − 80 °C for subsequent electron microscopy and proteomics analyses or stored at 4 °C for less than 1 week for use in functional assays.

The human breast cancer cell line MDA-MB231 [45] was cultured in RPMI (Corning) containing 10% human AB serum (Corning), 2 mM l-glutamine (Corning), 100 IU penicillin, and 100 μg/ml streptromycin (Corning) in a 37 °C incubator with 5% CO_2_. The MCF10DCIS.com cell line was cultured as previously described [46, 47]. The cells were tested every 3 months to confirm mycoplasma negativity (MycoAlert™ Mycoplasma Detection Kit, Lonza), and validated for authenticity by fingerprinting performed by Dr. Christopher Korch (University of Colorado Cancer Center Sequencing Facility). To make conditioned media, cells were grown to 80% confluency, rinsed with Hanks Buffered Saline Solution, and incubated at 37 °C in serum-free media for 4 h to minimize serum protein and EV contamination. Cells were then transferred to fresh serum-free media and incubated for 48 h at 37 °C. Cell debris was removed by centrifugation at 500×*g* for 5 min and 2000×g for 10 min. Supernatant was filtered through a sterile 0.22-μm syringe filter and stored at 4 °C. To isolate EVs, approximately 180 ml of conditioned media was concentrated to 1 ml by centrifugation in a 50-kDa molecular weight cutoff ultrafiltration tube (Sartorius) and isolated over a size-exclusion column as described above.

### Nanoparticle tracking analysis (NTA)

EV concentration and size were analyzed using a Nanosight NS300 instrument with a 532-nm laser (Malvern). Images were captured using an sCMOS camera, with a gain of 1.0, and camera level of 13. EVs purified by size-exclusion chromatography (SEC) were diluted 200-fold in phosphate-buffered saline (PBS) and injected using a Nanosight autopump (Malvern) in script mode commanding a set temperature of 22 °C, an infusion rate of 25 μl/min, and video capture of five consecutive 30-s videos with a 5-s delay. Data were captured and analyzed using NTA Analytical Software suite version 3.1 (Malvern) with a detection threshold of 5.0. The instrument was calibrated using 100 nm silicone beads. Samples that were below 20 particles per frame or above 100 particles per frame were re-diluted to a concentration within this range.

### Electron microscopy

EVs purified by SEC were incubated on formar-coated grids and negatively stained using 5% uranyl acetate. The grids were rinsed, and the size and morphology of EVs analyzed using a Technai 10 Transmission Electron Microscope (Field Emissions Inc.). Images were captured at 25,000× using a First Light digital camera (Gatan) (CU AMC Electron Microscopy Center, Aurora, CO).

### Western blots

Western blots were performed by separating 20 μg of protein in 1× RIPA buffer by 10% SDS-PAGE. Samples treated with 2.5 mU peptide-*N*-glycosidase F (Sigma Aldrich) were incubated for 3 h at 37 °C prior to SDS-PAGE separation. Protein bands were transferred to polyvinylidene fluoride (PVDF) membranes by wet transfer at 100 V for 1 h. The membranes were blocked with 5% non-fat dry milk in TBST and 10% goat serum and incubated with primary antibodies (Hsp70, CD81, CD63, CD9, System Biosciences) at 4 °C overnight. The membranes were washed in a mixture of tris-buffered saline and polysorbate 20 (TBST) and incubated in goat-anti rabbit IgG-horseradish peroxidase (HRP) secondary antibody (Systems Biosciences) at room temperature for 1 h. The protein bands were visualized using the ECL Plus Substrate solution (Pierce) and imaged using an Odyssey instrument (Licor Biotechnology).

### Sample preparation for proteomics

EV samples purified by SEC were analyzed via mass spectrometry (CU AMC Mass Spectrometry and Proteomics Shared Resource, Aurora, CO). The samples were digested according to the FASP protocol using a 30-kDa molecular weight cutoff filter [48]. In brief, samples were mixed in the filter unit with 8 M urea in 0.1 M ammonium bicarbonate (ABC), pH 8.5 and centrifuged at 14,000×*g* for 15 min. The proteins were reduced by addition of 100 μl of 10 mM DTT in 8 M urea and 0.1 M ABC, pH 8.5; incubated for 30 min at room temperature; and centrifuged. Subsequently, 100 μl of 55 mM iodoacetamide in 8 M urea and 0.1 M ABC, pH 8.5 was added to the samples, incubated for 30 min at room temperature in the dark, and centrifuged. The pellets were washed three times with 100 μl 8 M urea in 0.1 M ABC, pH 8.5, then three times in 100 μl of 0.1 M ABC buffer. The pellets were digested overnight at 37 °C with 0.02% Protease Max (Promega). Peptides were recovered by transferring the filter unit to a new collection tube and spinning at 14,000×*g* for 10 min. To complete peptide recovery, the filters were rinsed twice with 50 μl 0.2% FA and 10 mM ABC and collected by centrifugation. The peptide mixture was desalted and concentrated on a C18 Tip (Thermo Scientific Pierce).

### Mass spectrometry

Samples were analyzed on a Q Exactive quadrupole orbitrap mass spectrometer (Thermo Fisher Scientific) coupled to an Easy-nLC 1000 UHPLC (Thermo Fisher Scientific) through a nanoelectrospray ion source. Peptides were separated on a self-made 15-cm C18 analytical column (100 μm × 10 cm) packed with 2.7 μm Phenomenex Cortecs C18 resin [49]. After equilibrations with 3 μl 5% acetonitrile and 0.1% formic acid, the peptides were separated by a 180-min linear gradient from 2 to 32% acetonitrile with 0.1% formic acid at 350 nl/min. LC mobile phase solvents and sample dilutions used 0.1% formic acid in water (buffer A) and 0.1% formic acid in acetonitrile (buffer B) (Optima™ LC/MS, Fisher Scientific). Data acquisition was performed using the instrument supplied Xcaliber™ (version 3.0) software. The mass spectrometer was operated in the positive ion mode and in the data-dependent acquisition mode. In one scan cycle, peptide ions were first scanned by full MS at resolution 60,000 (FWHM at m/z 200), and then, the top 12 intensive ions (2 m/z isolation window) were sequentially subjected to HCD fragmentation and detected at resolution 15,000. Dynamic exclusion was set to 20 s. Spray voltage was set to 2.5 kV, S-lends RF level at 55, and heated capillary at 275 °C.

### Protein identification

MS/MS spectra data were extracted from raw data files and exported as mascot generic format files (mgf) using MassMatrix. The mgf files were then searched against the SwissProt database using an in-house Mascot™ server (version 2.2.06, Matrix Science). Mass tolerances were ± 10 ppm for MS peaks and ± 0.1 Da for MS/MS fragment ions. Trypsin specificity was used, allowing for one missed cleavage. Methionine oxidation, proline hydroxylation, protein N-terminal acetylation, and peptide N-terminal pyroglutamic acid formation were allowed for variable modifications while carbamidomethyl of Cys was set as a fixed modification. All raw or processed data files are available upon request.

Scaffold (version 4.4, Proteome Software) was used to filter tandem MS-based peptide and protein identifications. Peptide and protein identifications were accepted if they could be established at greater than 95% and 99% probability, respectively, as specified by the Peptide Prophet algorithm. Protein identifications also required at least two identified unique peptides.

Resultant proteomic data from cell line-derived EVs were compared by overall enrichment scores using DAVID Bioinformatics Resource [50, 51]. Proteins with 6 or more spectral matches were included in the analysis, and enrichment scores greater than 1.5 were reported. Proteomic data from patient EVs, in which more than two groups were compared, were analyzed using online statistical software MetaboAnalyst 3.0 [52]. Data was normalized by sum, auto-scaled (mean-centered and divided by the standard deviation of each variable), and multivariate and statistical analyses such as *t* tests, volcano plots, and partial least squares discriminant analysis (PLS-DA) were performed. Normalized data was exported from MetaboAnalyst 3.0, and further statistical analyses were performed using GraphPad Prism 7.

### Tumor cell motility assays

ImageLock 96-well plates (Essen Bioscience) were coated with 0.2 mg/ml Matrigel diluted in 1× PBS (Corning Life Sciences) for 2 h at room temperature and rinsed twice with 1× PBS. MCF10DCIS.com cells were seeded at 4000 cells per well and incubated overnight to 100% confluency at 37 °C in low-serum culture media containing 1% horse serum, ± 5 × 10^8^ EVs, and ± 3 μM FAK inhibitor (PF-573.228, Sigma). For the migration assays, uniform scratch wounds were created in the center of each well using the IncuCyte Wound Maker (Essen BioScience). Cells were washed, incubated in low-serum media, and bright-field images were taken every 2 h using an IncuCyte ZOOM® live cell imaging instrument (Essen BioScience). After 24 h, images were analyzed using IncuCyte ZOOM® analysis software and the density of cells in each wound area was calculated. For the invasion assays, a 2-mg/ml Matrigel pad was layered over the cells after wounding and images were captured for 48 h [53]. Flow cytometry was performed on a BD Fortessa X-20 after staining cells treated as described above with antibodies specific for total FAK (Biolegend, clone W16060A) and phosphorylated FAK (Fisher Scientifics, clone 31H5L17).

### Multiplex gene expression analysis

MCF10DCIS.com cells were plated at 40,000 cells per well in 96-well plates and cultured overnight at 37 °C. EVs from YWBC patients, healthy donors, or MDA-231 cells were then added and incubated for 18 h. Cells were then trypsinized, washed, and RNA isolated using a NucleoSpin RNA isolation kit according to the manufacturer’s instructions (Macherey-Nagel). RNA expression of genes related to cancer pathways (PanCancer Cancer Pathways Panel, #XT-CSO-PATH1) and cancer progression (PanCancer Progression Panel, #XT-CSO-PROG1) were measured using NanoString technology. Data were normalized and analysis performed using the NanoString nSolver 3.0 software to conduct nCounter advanced analysis (NanoString Technologies, Seattle, WA, USA).

### Statistical analysis

Two groups were compared using an unpaired two-tailed Student’s *t* test, three or more groups were compared using one-way ANOVA, and three or more groups with multiple measures were compared by two-way ANOVA using GraphPad Prism software version 6.0. Where appropriate, *p* values are adjusted for multiple comparisons and multiple measurements.

## Results

### Invasive breast cancer EVs increase the motility of less aggressive breast cancer cells

**Fig. 1:**
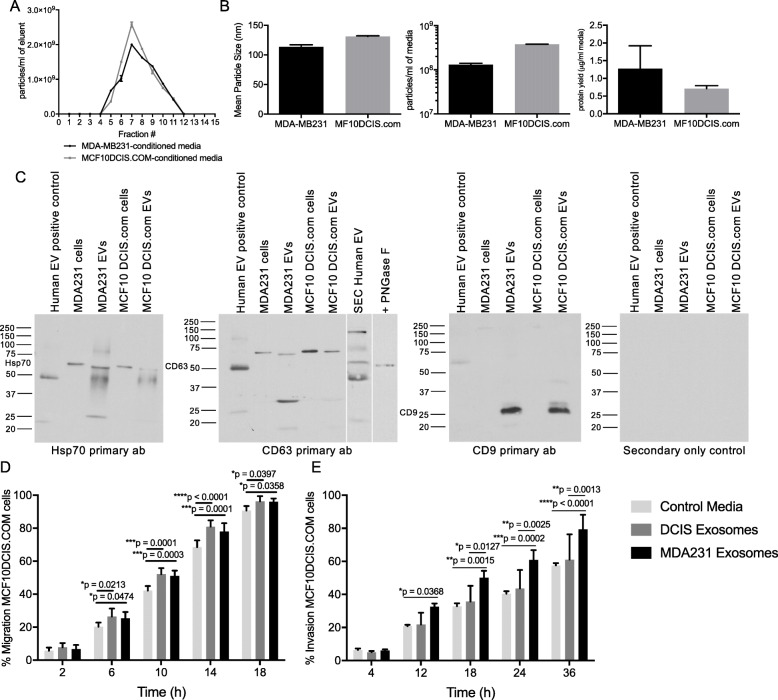
EVs increase the motility and invasion of breast cancer cells. **a** MDA-MB231 and MCF10-DCIS.com cells were isolated by size-exclusion chromatography (SEC). Fractions 5–10 were combined and characterized by NTA and BCA assay (**b**) and western blot (**c**). Human EV positive control was purchased from System Biosciences, consisting of EVs purified using Exoquick (System Biosciences). To demonstrate the effects of glycosylation, SEC-purified human EVs were treated with *N*-glycosidase F and blotted for CD63 primary ab. The lower bands (~ 25 kDa) in Hsp70 and CD63 blots are likely non-specific. **d** MCF10-DCIS.com cells were seeded ± 5 × 10^8^ EVs in 96-well plates coated with 0.5 mg/ml Matrigel, and phase contrast images were taken over 96 h using an IncuCyte instrument after wounding. **e** For the invasion assays, cells were plated as in **d** and covered with a 2-mg/ml Matrigel pad after wounding. Cell invasion was determined ± 5 × 10^8^ EVs over 36 h using an IncuCyte instrument. Averages of at least 4 independent experiments, each with 4 to 5 replicate wells, are shown. Groups were compared using two-way ANOVA, and *p* values were adjusted for multiple repeated measures. Error bars represent the standard deviation of the mean

To determine if the proteomic content of EVs correlates with function and could potentially serve as a biomarker of a breast cancer patient’s disease state, we first compared the proteomic content and function of EVs from two representative breast cancer cell lines differing in metastatic attributes. We characterized EVs secreted by the MDA-MB231 breast cancer line, aggressive triple negative breast cancer cells that form invasive carcinomas in xenograft models and which have been demonstrated to produce EVs that increase the motility of less invasive breast cancer lines [45, 54]. The second cell line used was MCF10DCIS.com cell line, which readily forms DCIS-like tumors and can become invasive in some conditions [47, 55]. EV characteristics of this cell line have not been previously described. We found that the physical properties of EVs from these breast cancer cell lines were similar to each other and to previously reported studies of EVs. Specifically, both cell lines produced approximately 100 nm particles that eluted between fractions 5 and 11 of the size-exclusion column (column volumes 5 ml through 11 ml, Fig. 1a) and had similar average particle sizes and protein concentrations (Fig. 1b) consistent with previous reports for MDA-MB231 EVs (Fig. 1b, [56]). These EVs also expressed the putative EV proteins Hsp70, CD63, and CD9 by western blot (Fig. 1c). These proteins are detected at multiple molecular weights, likely due to their known isoforms and differential glycosylation patterns [57–60]. In fact, treatment of EV lysate with *N*-glycosidase F reduced CD63 to a single detectable band at the reported molecular weight (Fig. 1c). Consistent with previous studies, EVs produced by invasive MDA-MB231 cells significantly increased the migration and invasion of MCF10DCIS.com cells across a scratch wound (Fig. 1d, e). In contrast, EVs produced by the less aggressive MCF10DCIS.com cells did not increase invasion of MCF10DCIS.com cells but did increase migration in the scratch wound assay. There was no significant effect of EVs from either cell line on the motility of MDA-MB231 cells in these assays (data not shown), suggesting that these cells are already maximally invasive.

### The protein content of EVs from breast cancer cell conditioned media is consistent with their function and cell of origin

The differential effect of EVs from invasive and non-invasive breast cancer cells on the motility and invasive capabilities of the DCIS.com cells suggests there may be proteomic differences between EVs isolated from these cell lines. To address this question, we compared the total protein content of EVs isolated from two independent preparations of conditioned media from MDA-MB231 and MCF10DCIS.com cells by mass spectrometry. Shared proteins identified in EVs from both cell lines were enriched for vesicle proteins (enrichment score of 7.4), membrane components (2.97), regulators of cell death (2.86), contractile fibers (2.56), adhesion molecules (1.89), and proteins that promote cell motility (1.84), and included putative EV proteins such as Hsp70, CD63, and CD9. EVs produced by the invasive MDA-MB231 cells were significantly enriched for proteins involved in vesicle formation (enrichment score of 6.16), protein synthesis (4.9), proteolysis (3.56), and glycolysis (1.54). In contrast, MCF10DCIS.com EVs were significantly enriched for membrane proteins (12.65 enrichment score), adhesion molecules (10.33), proteins involved in cellular migration (4.21), and components of the extracellular matrix (3.65). Reflecting these differences, the most abundant proteins uniquely identified in MDA-MB231 EVs were those involved in transcriptional regulation (splicesome, transcription factors, ribosomal proteins, tRNA ligases), proteolysis (proteasome units, pyrophosphatase), EV formation (annexin and vesicle markers LAMP-1 and EEA1), cell cycle (NUMA1), and cell motility and adherence to extracellular matrices (vitronectin, collagen, filamin proteins, and EDIL3) (Table 1). In contrast, the most abundant proteins uniquely identified in EVs from the MCF10DCIS.com cells were cellular adhesion proteins (cadherin family members, laminin proteins, proteoglycans, syndecan-1, EPCAM, b-catenin, and collagen), regulators of cellular proliferation (CD109, RARRES1, PTGFRN, FAT1, S100A14, and amphiregulin), and metabolic proteins (calcium-binding proteins, serine proteases, and cholesterol- and lipoprotein-binding proteins) [61, 62]. These results suggest that the protein content of EVs from MDA-MB231 and MCF10DCIS.com cells reflects the biologic differences between these invasive and non-invasive breast cancer cells and thus may contribute to their altered functional activity.

**Table 1:** Abundant proteins identified uniquely in MDA-MB231 or MCF10DCIS.com EVs purified from cell culture supernatants

MDA-MB231 EVs		MCF10DCIS.com EVs	
Protein name	No. of spectral matches^A^	Protein name	No. of spectral matches^A^
Complement C4-B	140.2	Protocadherin fat 2	264.6
EGF-like repeat and discoidin I-like protein 3	101.7	Laminin subunit beta-3	238.6
Vitronectin	101.2	Chondroitin sulfate proteoglycan 2	200.7
Annexin A6	88.5	CD109 antigen	196.8
NUMA1 variant protein	88.3	Retinoic acid receptor responder protein 1	189.0
Filamin-C	50.3	Prostaglandin F2 receptor negative regulator	160.1
Thioredoxin reductase 1	47.5	Laminin, alpha 4	147.3
Collagen, type V, alpha 1	44.4	Syndecan-1	139.9
Proteasome subunit beta type-4	39.4	Protocadherin fat 1	110.0
Filamin-A	38.2	Stromal cell derived factor 4	108.3
U5 small nuclear ribonucleoprotein	37.9	Epithelial cell adhesion molecule	105.3
Early endosome antigen 1	37.6	Protein S100-A14	97.3
Ectonucleotide pyrophosphatase	36.2	SPARC related modular calcium binding 1	83.9
Proteasome subunit alpha type-1	32.4	Fibulin-1	68.9
Interleukin enhancer-binding factor 2	30.8	Suppressor of tumorigenicity 14 protein	65.6
60S ribosomal protein L12	28.7	Catenin, beta 1	59.4
Ubiquitin-like protein ISG15	27.8	Serpin peptidase inhibitor, clade E	56.1
Aspartate-tRNA ligase	25.6	Follistatin	48.0
Proteasome subunit beta type 7	24.5	Collagen alpha-1	45.2
Serum paraoxonase/arylesterase 1	23.1	Claudin	45.1
26S proteasome non-ATPase subunit 6	22.1	Amphiregulin	44.0
Lysosomal-associated membrane protein 1	21.5	Lipolysis-stimulated lipoprotein receptor	43.8
Ferritin light chain	20.8	Fascin	42.5
26S protease subunit 8	20.6	Plakophilin-3	41.5
60S ribosomal protein L27	19.8	Isoform 4 of scavenger receptor class B1	40.5
SARS protein	19.3	Solute carrier 16, member 1	37.1

Of the 1109 proteins identified in MCF10DCIS.com EVs and the 1032 proteins identified in MDA-MB231 EVs, 632 proteins were shared between the two cell lines

^A^Spectral matches are the averages of two independent EV preparations

### EVs from YWBC patients increase breast cancer cell invasion

**Fig. 2:**
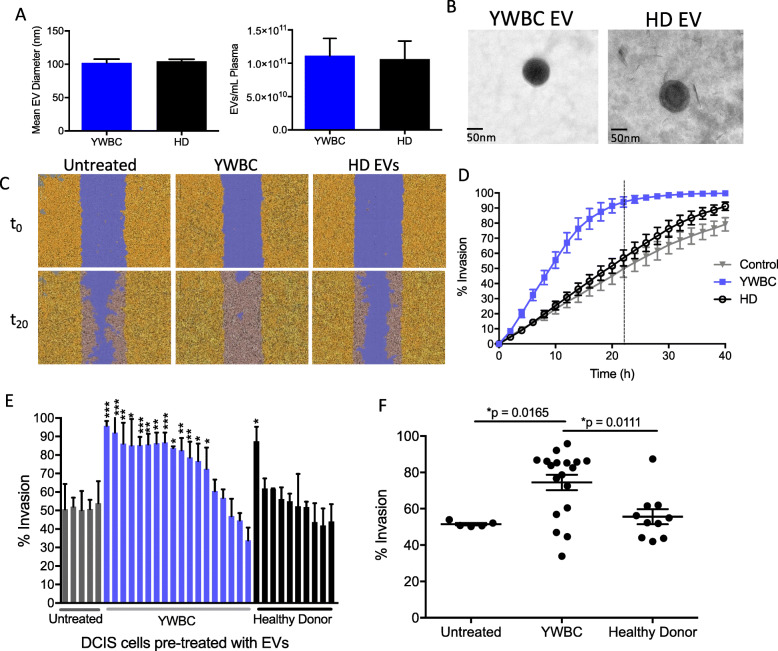
EVs isolated from YWBC patients promote increased invasion of MCF10DCIS.com breast cancer cells. EVs isolated from human YWBC patients or healthy donor (HD) plasma samples by size-exclusion chromatography were incubated with MCF10DCIS.com human breast cancer cells in a scratch wound assay overlaid with a Matrigel pad. Phase images were taken every 2 h using an IncuCyte instrument. After 48 h, images were analyzed using IncuCyte ZOOM software. **a** Mean diameter and number of EVs isolated from YWBC plasma or HD plasma. **b** Electron microscope images of YWBC EV and HD EV. **c** Representative images of the invasion assay showing the initial scratch wound (*t*_0_) and after 20 h (*t*_20_). The percentage invasion at each time point was calculated from the density of cells in each wound (shown in yellow) relative to the initial wound (shown in purple). **d** The average percent invasion of 4 replicate wells was plotted over time. Representative data for untreated MCF10DCIS.com cells (gray) or treated with EVs from a YWBC patient (blue) or HD (black). **e** The average percent invasion at the time point when untreated controls reached 50% confluence was compared to individual treatments using Student’s *t* test (left). The average percent invasion for each condition was compared using one-way ANOVA with a multiple comparisons test (right). There was no significant difference between untreated cells and those treated with EVs from healthy donors

Since the largest functional differences were observed in the 2D scratch wound invasion assay through the Matrigel pad (Fig. 1e), we next determined whether EVs isolated from the peripheral blood of human YWBC patients increased invasion in breast cancer cells compared to EVs from healthy donors. EVs from 18 YWBC patients and 10 healthy donors (Additional file 1) were isolated using size-exclusion chromatography. Similar to EVs isolated from the breast cancer cell lines, the majority of small particles from both YWBC patients and healthy donors eluted in fractions 5 through 10 (Additional file 2). The average particle size and yield were similar between YWBC patients and healthy donors (Fig. 2a), and isolated EVs had a classically spherical cup-shaped appearance by electron microscopy (Fig. 2b). Incubation with EVs from YWBC patients significantly increased the density of MCF10DCIS.com cells inside scratch wounds compared to untreated controls or EVs from healthy donors (Fig. 2c). To compare across multiple patients and assays, the average percent invasion of experimental conditions was compared at the time point in which the untreated controls reached 50% invasion (Fig. 2d). Compared to the corresponding untreated controls, incubation with EVs from 13 of the 18 YWBC patients and only 1 of the 10 healthy donors significantly increased MCF10DCIS.com invasion over untreated control cells (Fig. 2e). On average, EVs from YWBC patients significantly increased MCF10DCIS.com cell invasion (74.5% wound closure) over that of untreated controls (50.2% wound closure) or cells treated with EVs from healthy donors (55.6% wound closure) (Fig. 2f). Functional EVs were identified in patients across risk factor subsets, including subtype, stage, body mass index, and parity (Additional file 3).

### EVs from YWBC patients have a unique proteome compared to EVs from healthy donors

The functional effects of EVs from YWBC patients on breast cancer cell invasion suggest that their protein content might be distinct from that of healthy donors. To elucidate the specific differences between healthy and breast cancer-associated human EVs, we compared the proteomic content of EVs isolated from 20 YWBC patients to that of EVs isolated from 10 healthy donors (Additional file 1) using a simple top-down proteomic approach [63].

**Fig. 3:**
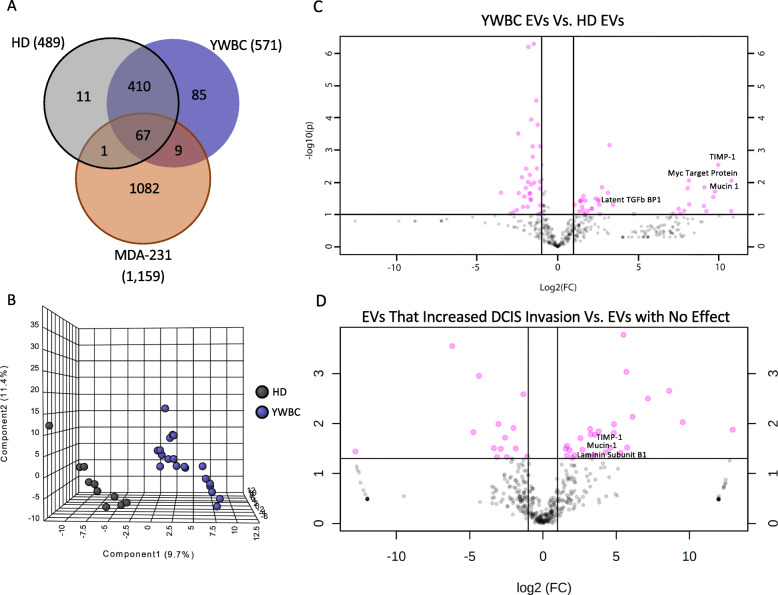
EVs from YWBC patients have a unique proteomic signature. EVs from 20 YWBC patients and 10 healthy donors were purified by size-exclusion chromatography and analyzed by mass spectrometry. The identified proteins were analyzed using MetaboAnalyst 3.0 software. **a** Of the 583 proteins identified, EVs from YWBC patients contain 94 unique proteins compared to EVs from healthy donors. **b** Multivariate analysis was performed using the partial least squares discriminant analysis method. Variables were sorted into components according to their ability to discriminate between YWBC patients and healthy donors (HD). The 3D plot of component 1 (*A* = 0.83, *R*^2^ = 0.82, *Q*^2^ = 0.50), component 2 (*A* = 0.97, *R*^2^ = 0.82, *Q*^2^ = 0.71), and component 3 (*A* = 0.96, *R*^2^ = 0.97, *Q*^2^ = 0.64) is shown, in which the number of spectral matches for proteins in these groups distinguishes YWBC (blue) from HD (black). A volcano plot analysis was performed using MetaboAnalyst 3.0, comparing the proteomic differences between EVs from YWBC patients and HD (**c**) or EVs with functional activity in Fig. 2 and those with no effect (**d**). Fold change values are represented on the *x*-axis, and *t* test *p* values are represented on the *y*-axis (see Additional file 4 for specifics). Proteins of interest for breast cancer are labeled

Of the 571 proteins identified, YWBC EVs contain 85 unique proteins, 76 proteins that overlap with MDA-MB231 EVs (Fig. 3a), and 70 proteins that overlap with MCF10DCIS.com EVs. We consistently identified typical EV markers CD9, CD81, CD63, and HSP70, and proteins vital for EV formation such as Rab proteins, tetraspanins, clatherin components, and myosin proteins in EVs from both healthy donors and YWBC patients [1]. Proteins unique to YWBC EVs and EVs from the cell lines that were not identified in HD EVs include serpin B3, tripeptidyl-peptidase 2, prolactin-inducible protein, tetraspanin-15, and proteasome subunits (Additional file 4).

To compare EVs from YWBC and healthy donors, multivariate analysis using the partial least squares discriminant analysis method was performed and variables were sorted into principal components based on their ability to discriminate between YWBC and healthy donor EV content (Fig. 3b). Volcano plot analysis identified 46 proteins that significantly differ between YWBC and healthy donor EVs with a fold change threshold of 0.2 and a *p* value threshold of 0.05 (Fig. 3c, Additional file 4). Of interest in breast cancer, EVs from YWBC patients had increased levels of Mucin 1, TIMP-1, Myc Target Protein, and Latent TGFB binding protein1. Further, proteins such as Mucin 5b, Mucin 1, TIMP1, and Laminin B1 were significantly enriched not only in breast cancer EVs as compared to healthy donor EVs, but also specifically in the EVs that increased invasion compared to those that had no effect on invasion (Fig. 3d).

### EVs from invasive breast cancer cells and YWBC patients alter gene expression in treated cells

**Fig. 4:**
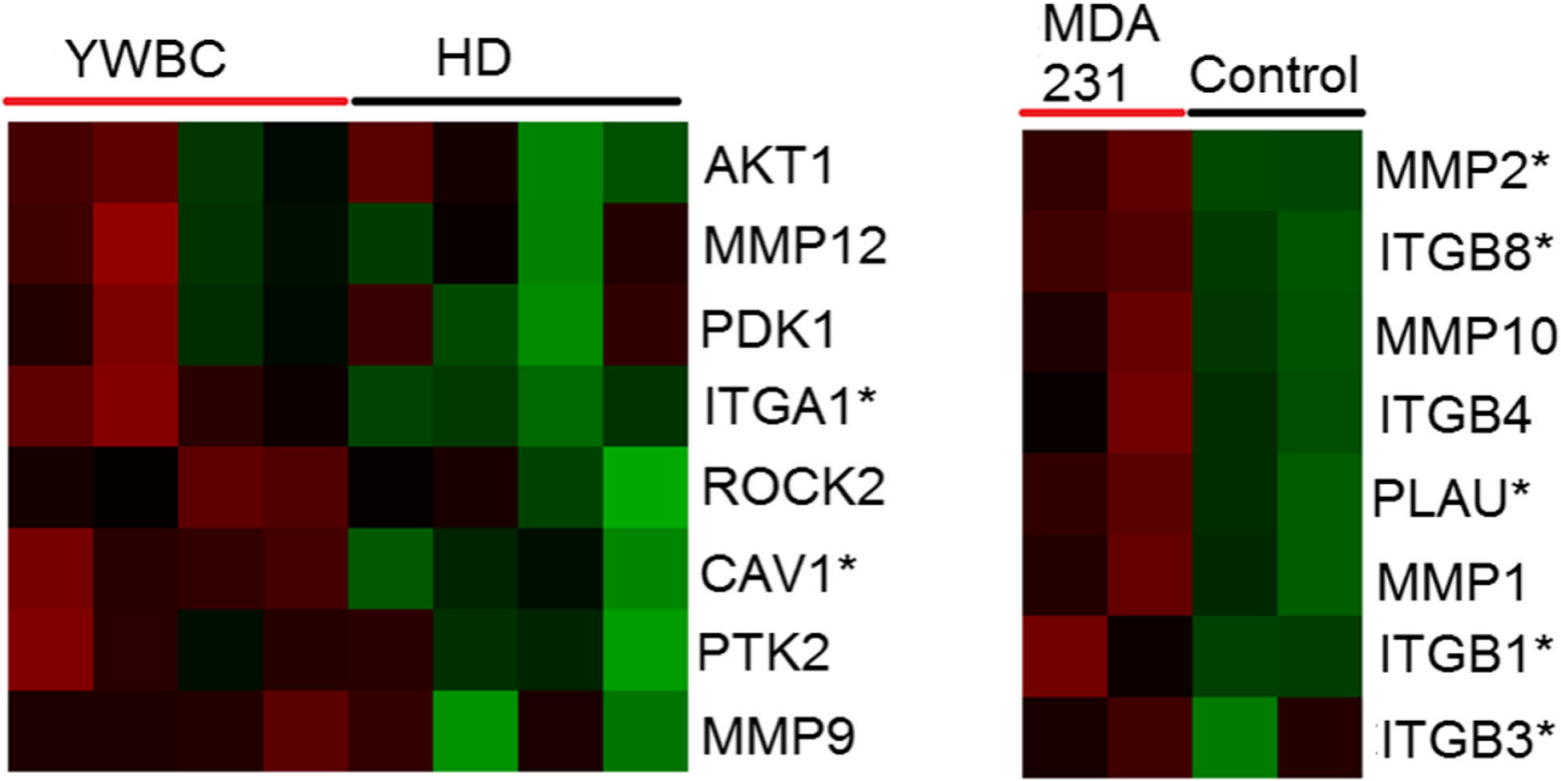
EVs alter expression of genes in the FAK pathway in treated MCF10DCIS.com breast cancer cells. MCF10DCIS.com cells were incubated with EVs from YWBC patients, healthy donors (HD), or MDA231 breast cancer cells for 18 h prior to RNA isolation. Gene expression related to cancer pathways and cancer progression was measured using NanoString technology. Data were normalized and statistical analysis performed using the NanoString nSolver3.0 analysis software. Heatmap of the top 8 genes related to the FAK pathway comparing MCF10DCIS.com cells treated with EVs from YWBC patients to HD (left) or from invasive MDA231 breast cancer cells to untreated controls (right), *p < 0.05

We next sought to determine whether cancer cell signaling pathways are altered after treatment with EVs. We compared gene expression in MCF10DCIS.com cells treated with EVs isolated from YWBC patients, healthy donors, invasive MDA-MB231 cells, and untreated cells using NanoString analysis of the Cancer Pathway and Cancer Progression gene sets. nSolver and nCounter analysis identified significantly altered expression of genes related to cell motility and EMT, cell adhesion, angiogenesis, and proliferation (Table 2). KEGG pathway analyses revealed EV-induced alterations of the Focal Adhesion Kinase (FAK) pathway. This was supported by our proteomics above, which identified EV-associated proteins that have demonstrated involvement in FAK-mediated cell motility (tetraspanin-15, proteasome subunits) [64–66]. These proteins were identified exclusively in YWBC and MDA-MB231 EVs, but not in healthy donor EVs, suggesting a potential role of the FAK pathway in the functionality of these EVs (Additional file 4). Additionally, a number of proteins known to upregulate the FAK pathway were significantly enriched in EVs that increased cell invasion in our functional assays (TIMP1, Mucins, and Laminin B1). This led us to further investigate the role of the FAK pathway in driving EV-induced invasion. As shown in Fig. 4, several genes in the FAK pathway were significantly altered after treatment with breast cancer EVs.

**Table 2:**
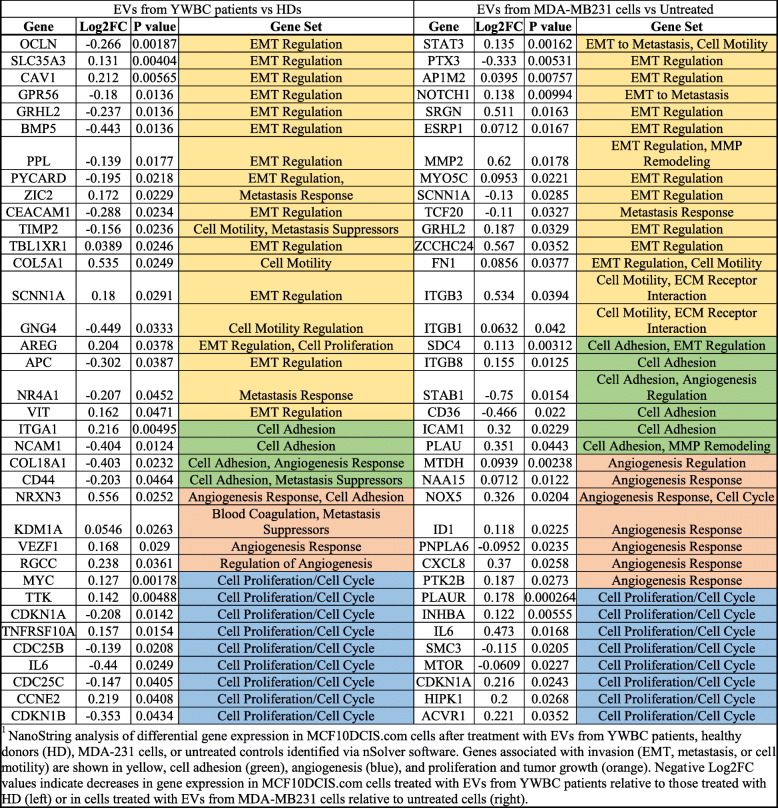
EVs alter expression of genes related to cancer progression in treated MCF10DCIS.com breast cancer cells

NanoString analysis of differential gene expression in MCF10DCIS.com cells after treatment with EVs from YWBC patients, healthy donors (HD), MDA-231 cells, or untreated controls identified via nSolver software. Genes associated with invasion (EMT, metastasis, or cell motility) are shown in yellow, cell adhesion in green, angiogenesis in blue, and proliferation and tumor growth in orange. Negative log2FC values indicate decreases in gene expression in MCF10DCIS.com cells treated with EVs from YWBC patients relative to those treated with HD (left) or in cells treated with EVs from MDA-MB231 cells relative to untreated cells (right)

### Inhibition of FAK pathway signaling attenuates EV-induced increases in breast cancer cell invasion

**Fig. 5:**
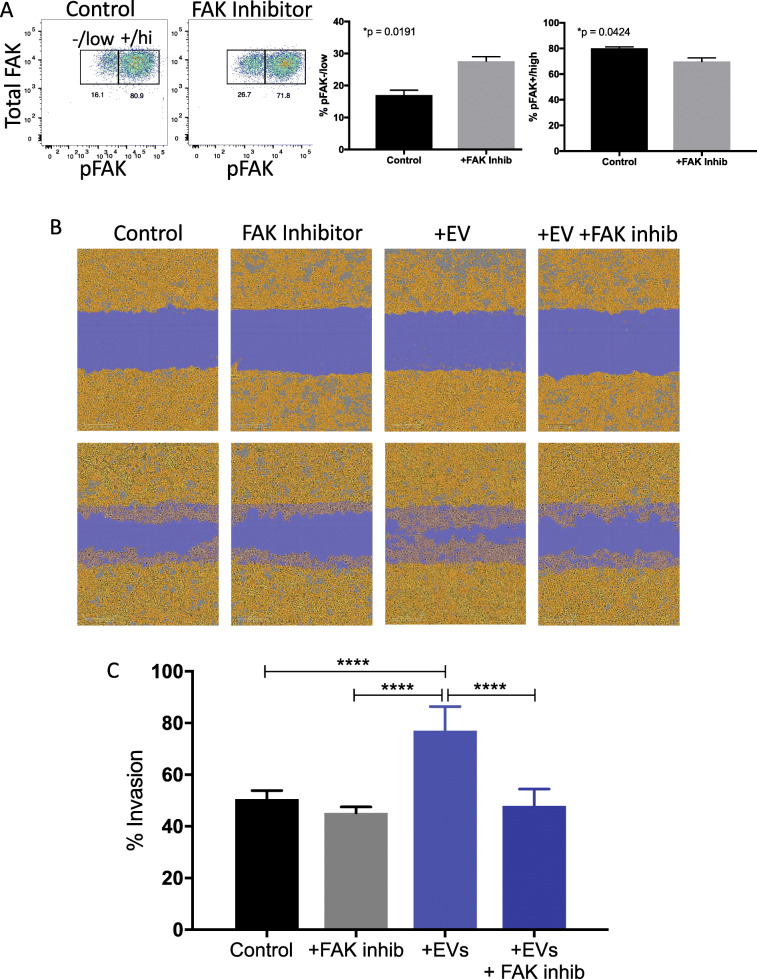
FAK pathway inhibition attenuates increased invasion after EV treatment. Invasion assays were performed and analyzed as described above treating MCF10DCIS.com cells with EVs isolated from MDA.MB231 cells in the presence and absence of a FAK inhibitor (PF.573.228). **a** Incubation with FAK inhibitor decreases phosphorylated FAK protein as detected by flow cytometry. **b** Representative images of cell densities (yellow) overlaid with initial scratch wounds (purple). **c** The average percent invasion of 4 replicate wells containing MCF10DCIS.com cells treated with EVs ± 3 μM FAK inhibitor

Since proteomic and genomic analysis implicated the FAK pathway as a potential driver of the functional effects of breast cancer EVs, we hypothesized that inhibition of the FAK signaling pathway using a validated inhibitor (PF-573.228) may reduce EV-induced breast cancer cell invasion. PF-573.228 is an established small molecule inhibitor that has a 50- to 250-fold selectivity for FAK over other protein kinases [67]. To determine whether inhibition of FAK affects breast cancer cell invasion, we performed the invasion assay with or without EV treatment and in the presence or absence of the FAK inhibitor. Incubation with the FAK inhibitor decreased FAK phosphorylation, detected by flow cytometry (Fig. 5a). As reported above, treatment with EVs from MDA-MB231 cells increased the invasion of MCF10DCIS.com cells through a Matrigel pad (Fig. 5b, c). Although FAK inhibition in the absence of EVs did not affect invasion, the FAK inhibitor significantly abrogated the increase in EV-stimulated DCIS.com cell invasion to levels matching the untreated control (Fig. 5b, c).

## Discussion

Circulating EVs hold the promise to provide a source of relevant biomarkers for breast cancer onset and recurrence, an important advancement for early detection and post-treatment surveillance of breast cancer patients. EVs secreted by breast cancer cells have been shown to have functional consequences on their surrounding environment and at distant sites of metastasis and therefore also represent potential novel targets for therapeutic development [30, 40, 68]. In this study, we hypothesized that we would detect proteomic and functional differences between EVs isolated from patients with YWBC versus those isolated from age-matched healthy donors.

We first confirmed that EVs derived from aggressive breast cancer cells influence the invasive behavior of a normally non-invasive breast cancer cell line. EVs produced by the invasive triple negative MDA-MB231 breast cancer cell line increased both the migration and invasion of MCF10DCIS.com cells in scratch wound assays. These results support previous studies showing that EVs produced by highly invasive breast cancer cells, and specific proteins enriched in EVs, can increase the growth and metastatic potential of more indolent breast cancer cells [68–71]. For example, EVs isolated from breast cancer cell lines contain metalloproteases with catalytic activity that increase the migration of less aggressive breast cancer lines [38, 56, 72] and may contain EGF ligand and microRNA that contribute to increased tumor cell invasion [61, 73]. Additionally, EVs have been implicated in cancer drug resistance, as they have been shown to sequester cytotoxic drugs and/or deliver mRNA, microRNA, and proteins that induce chemoresistance [74, 75]. In light of these studies, EVs may have a pronounced importance for the detection and treatment of breast cancer.

We next demonstrated that EVs derived from YWBC patients increased the invasive behavior of DCIS.com cells compared to cells treated with EVs from healthy donors or untreated controls. Furthermore, we determined that YWBC EVs have a unique proteomic content compared to EVs isolated from age-matched healthy donors that may facilitate the observed functional effects. In this study, both Mucin 1 and Mucin 5B were enriched in YWBC EVs. Mucins have been implicated in the induction of EMT, which can lead to cancer cell motility and metastasis, and have been established as candidate breast cancer biomarkers [54, 76]. Additionally, proteins involved in the c-MYC and TGF-β pathways were found in higher quantities in YWBC patient EVs, both of which are well established in the development and progression of breast cancer [77–81]. Interestingly, proteins such as Mucins, TIMP1, and Laminin B1 were specifically enriched in EVs that increased cancer cell invasion. Furthermore, proteins such as tetraspanin-15, prolactin-inducible protein, and proteasome subunits were identified specifically in EVs from YWBC patients and MDA-MB231 cells. These proteins are known to positively regulate the FAK signaling pathway, potentially leading to increases in cell motility [54, 64–66, 77, 82–84].

Gene expression analysis of breast cancer cells revealed alterations in gene expression patterns after treatment with EVs from YWBC patients and MDA-MB231 cells consistent with their increased motile and invasive phenotypes. A variety of genes involved in pathways related to cell motility, EMT, and metastasis were significantly altered, along with those related to cell adhesion, angiogenesis, and cell cycle regulation. EV-induced changes in the regulation of EMT, cell motility, and cell adhesion are likely related to our observed changes in the cell invasion assays, as cells must detach from neighboring cells, degrade their local matrix, and activate motility pathways in order to invade [85, 86]. Strikingly, treatment with EVs from YWBC patients and MDA-MB231 cells led to alterations in genes related to the FAK pathway, paralleling the FAK-related functions of many proteins identified in the proteomic analysis of the EVs themselves.

The FAK signaling pathway is activated by clustering of integrin receptors upon interactions with extracellular matrix (ECM) proteins, causing FAK dimerization and subsequent autophosphorylation [33, 35]. The FAK pathway promotes cell motility and invasion by regulating matrix metalloproteinase (MMP) expression, focal adhesion turnover, and actin cytoskeletal dynamics [33, 87]. Due to the potential role of FAK in cancer progression, a variety of inhibitors have been developed to target this molecule as a treatment for various cancers [33, 88]. Furthermore, inhibition of FAK significantly abrogated the EV-induced increased invasion to levels similar to untreated controls. Combined, these data implicate the FAK pathway as an important player in the pathologic effects of breast cancer EVs, as demonstrated here in our cohort of YWBC patients. While FAK inhibition has been studied as a potential treatment for breast cancer, the relationship of this signaling pathway and EV content and function has not previously been demonstrated.

One limitation of this study is that we have not determined whether the proteomic and functional changes in EVs isolated from our cohort of YWBC patients would also be identified in breast cancer patients of all ages. Further, we specifically included only nulliparous heathy donors as a comparator in this study and cannot exclude the possibility that parity may have contributed to some of the observed differences between healthy donors and YWBC patients. However, functional EVs were isolated from patients with various hormone receptor status, stage of disease, parity status, and BMI, suggesting that malignancy is the dominant contributor to the observed effects of EVs. Future studies in larger cohorts will determine whether the presence of specific proteins known to influence the FAK signaling pathway in circulating EVs from YWBC and postmenopausal breast cancer patients is related to age of diagnosis, disease state, parity status, clinical outcomes, or response to treatment. Finally, our study focuses on the response of triple negative breast cancer cell lines. It therefore remains to be determined whether EVs from breast cancer patients would have similar effects on breast cell lines expressing hormone receptors.

## Conclusions

This study not only reports the protein content and transformative effects of EVs isolated from YWBC patients for the first time, but also identifies signaling pathways in breast cancer cells that are affected by treatment with EVs. Taken together, these results suggest that circulating EVs from YWBC patients contain biologically relevant cargo that alter the behavior of cancer cells and may influence disease progression. Further, these EVs contain a unique set of proteins that could potentially serve as cancer biomarkers, and others that may be potential targets for individualized cancer treatment. This information, in combination with future studies involving added subsets of breast cancer, could also allow for the development of EV-targeted therapies for the treatment of breast cancer.

## Supplementary Information

**Additional file 1.** Clinical Characteristics of Enrolled Patients, study subject information.

**Additional file 2.** Isolation of human EVs from plasma of healthy donor and breast cancer patients, results of the size exclusion chromatography separation of EVs.

**Additional file 3.** EVs isolated from different subsets of YWBC patients increased invasion of MCF10DCIS.com breast cancer cells, clinical characteristics of functional EVs shown in Fig. 2.

**Additional file 4.** Proteins identified by volcano plot comparing EVs from healthy donors and YWBC patients, tabulated data of the volcano plot shown in Fig. 3.

## Data Availability

The datasets used and/or analyzed during the current study are available from the corresponding author on reasonable request.

## Abbreviations

DNA: Deoxyribonucleic acid
DTT: Dithiothreitol
ECL: Enhanced chemiluminescence
ECM: Extracellular matrix
EMT: Epithelial-to-mesenchymal transition
EV: Extracellular vesicle
FAK: Focal Adhesion Kinase
FWHM: Full width at half maximum
HRP: Horse radish peroxidase
MMP: Matrix metallopeptidase
MS/MS: Tandem mass spectrometry
NTA: Nanoparticle tracking analysis
PLS-DA: Partial least squares discriminant analysis
PVDF: Polyvinylidene difluoride
RNA: Ribonucleic acid
TBST: Tris-buffered saline polysorbate 20
SEC: Size-exclusion chromatography
YWBC: Young women’s breast cancer
