# Supplementary material for: Extracellular vesicles from young women’s breast cancer patients drive increased invasion of non-malignant cells via the Focal Adhesion Kinase pathway: a proteomic approach
Source: Breast Cancer Res. 2020 Nov 23;22:128. doi: 10.1186/s13058-020-01363-x (PMC7681773; doi:10.1186/s13058-020-01363-x)
Supplement: Supplementary file 1 — Additional file 1. Clinical Characteristics of Enrolled Patients, study subject information. [file 13058_2020_1363_MOESM1_ESM.pdf]

Additional File 1: Clinical Characteristics of Enrolled Patients

	Case	# of Patients	Age (Range)	Stage (# of Patients)
**Proteomics**	Luminal A	2	34 (33-35)	II (2)
	Luminal B	14	34 (27-40)	II(9), III(3), IV(2)
	Triple Negative	4	36 (29-40)	I(2), II(2)
	Healthy Donor	10	28 (23-48)	n/a
**Invasion Assays**	**Case**	**# of Patients**	**Age (Range)**	**Stage (# of Patients)**
	Luminal A	2	34 (33-35)	II (2)
	Luminal B	11	34 (27-40)	II(6), III(3), IV(2)
	Triple Negative	4	36 (29-40)	I(2), II(2)
	Healthy Donor	10	28 (23-48)	n/a
