# Supplementary material for: Extracellular vesicles from young women’s breast cancer patients drive increased invasion of non-malignant cells via the Focal Adhesion Kinase pathway: a proteomic approach
Source: Breast Cancer Res. 2020 Nov 23;22:128. doi: 10.1186/s13058-020-01363-x (PMC7681773; doi:10.1186/s13058-020-01363-x)
Supplement: Supplementary file 2 — Additional file 2. Isolation of human EVs from plasma of healthy donor and breast cancer patients, results of the size exclusion chromatography separation of EVs. [file 13058_2020_1363_MOESM2_ESM.pdf]

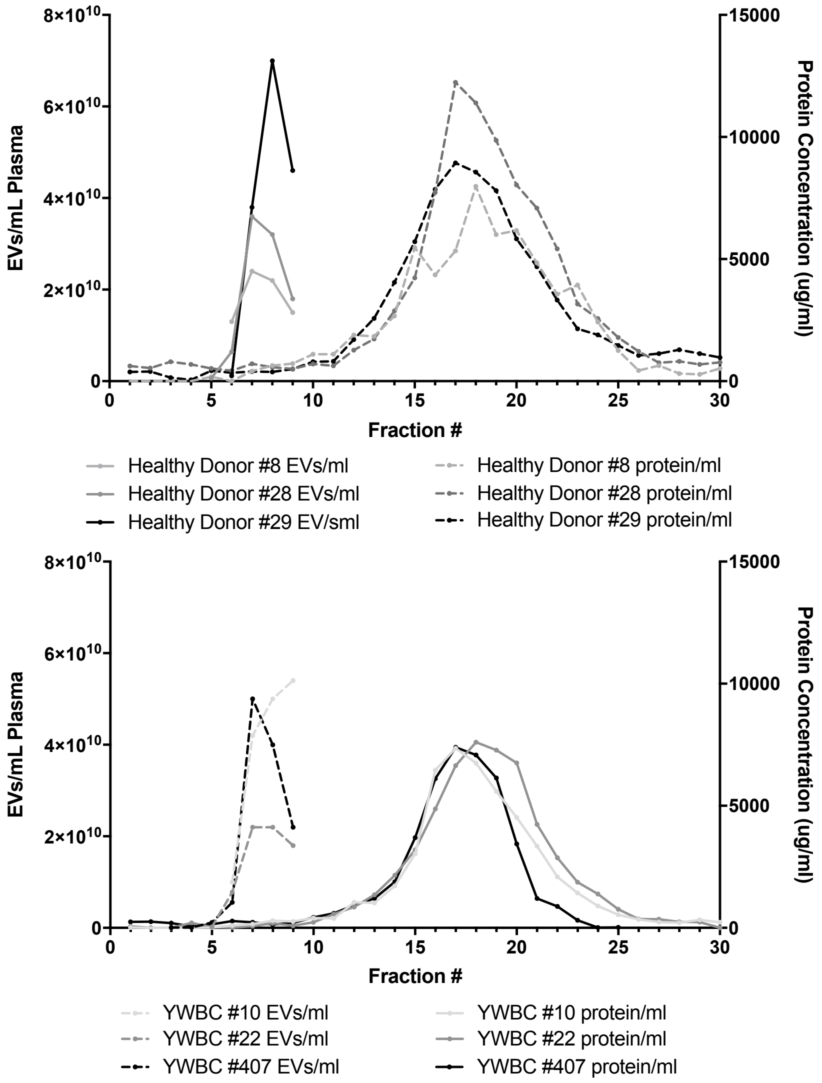

Additional File 2: Isolation of human EVs from plasma of healthy donor and breast cancer patients. Human plasma samples from healthy donors (top) or YWBC patients (bottom) were layered over a Sepharose CL-2B size-exclusion column (GE Healthcare) and 30 fractions were collected by gravity filtration. The EV content of each fraction was determined by NTA (solid lines, left axis) and the protein content was characterized by Bradford Assay (dashed lines, right axis).
