# Supplementary material for: Extracellular vesicles from young women’s breast cancer patients drive increased invasion of non-malignant cells via the Focal Adhesion Kinase pathway: a proteomic approach
Source: Breast Cancer Res. 2020 Nov 23;22:128. doi: 10.1186/s13058-020-01363-x (PMC7681773; doi:10.1186/s13058-020-01363-x)
Supplement: Supplementary file 3 — Additional file 3. EVs isolated from different subsets of YWBC patients increased invasion of MCF10DCIS.com breast cancer cells, clinical characteristics of functional EVs shown in Fig. 2. [file 13058_2020_1363_MOESM3_ESM.pdf]

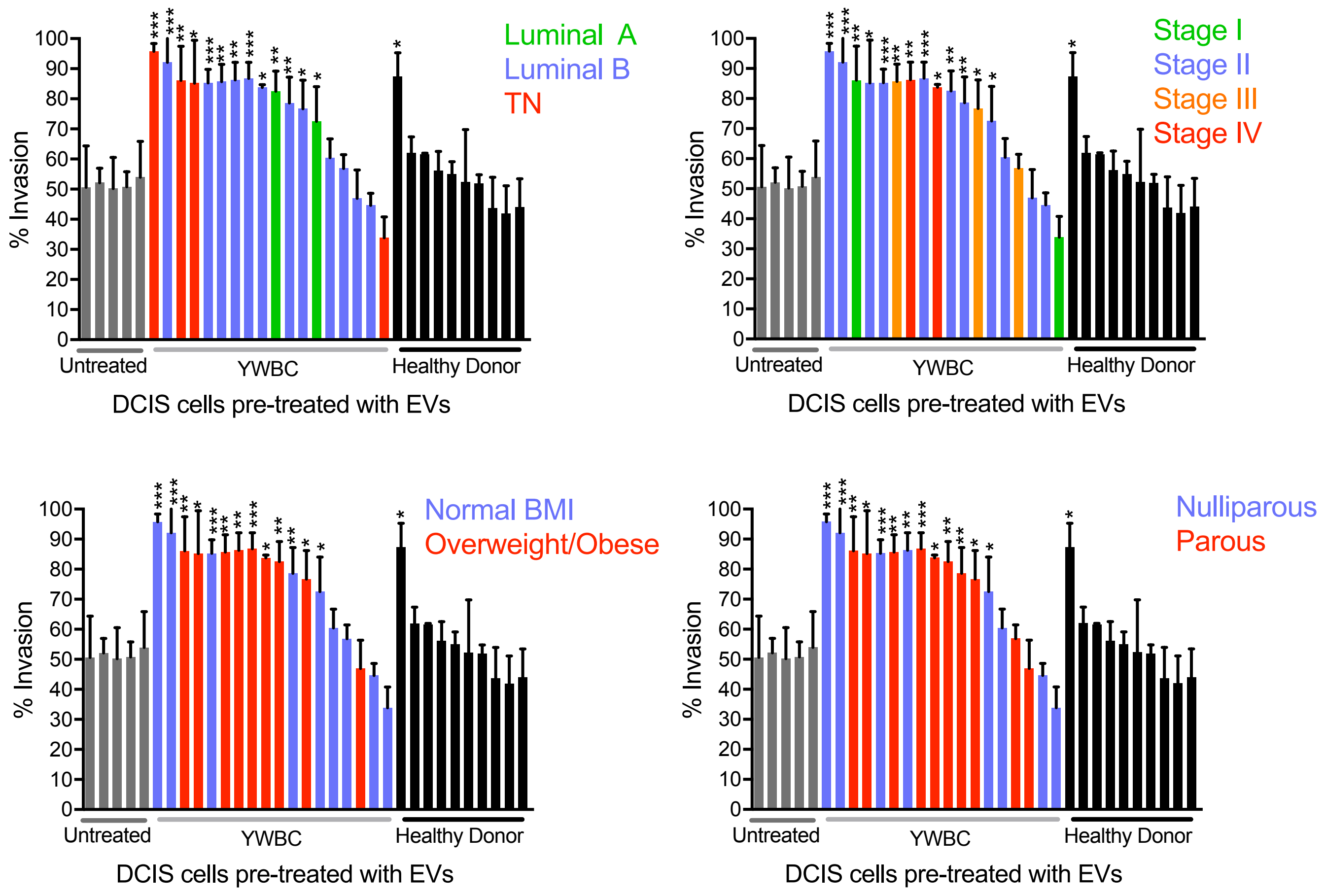

Additional File 3: EVs isolated from different subsets of YWBC patients increased invasion of MCF10DCIS.com breast cancer cells. EVs isolated from different subsets of human YWBC patients or healthy donor (HD) plasma samples by size exclusion chromatography were incubated with MCF10DCIS.com human breast cancer cells in a scratch wound assay as shown in Figure 2.
