# Supplementary material for: Extracellular vesicles from young women’s breast cancer patients drive increased invasion of non-malignant cells via the Focal Adhesion Kinase pathway: a proteomic approach
Source: Breast Cancer Res. 2020 Nov 23;22:128. doi: 10.1186/s13058-020-01363-x (PMC7681773; doi:10.1186/s13058-020-01363-x)
Supplement: Supplementary file 4 — Additional file 4. Proteins identified by volcano plot comparing EVs from healthy donors and YWBC patients, tabulated data of the volcano plot shown in Fig. 3. [file 13058_2020_1363_MOESM4_ESM.pdf]

Additional File 4: Proteins identified by volcano plot comparing EVs from healthy donors and YWBC patients and those shared with breast cancer lines.

Peaks(mz/rt)	FC	log2(FC)	p.value	-log10(p)
L-lactate dehydrogenase A chain	0.28673	-1.8023	0.00000	6.1971
Pyruvate kinase PKM	0.40268	-1.3123	0.00003	4.5333
Tropomyosin alpha-3 chain	0.32108	-1.639	0.00011	3.9497
Carbonic anhydrase 2	0.42568	-1.2321	0.00016	3.7846
Ras GTPase-activating-like protein IQGAP2	0.18458	-2.4377	0.00031	3.5121
Catalase	9.3408	3.2235	0.00070	3.1554
Cell division control protein 42 homolog	0.47393	-1.0773	0.00075	3.1231
Tropomyosin alpha-1 chain	0.34259	-1.5454	0.00077	3.1152
GMP reductase 1	0.34512	-1.5348	0.00161	2.7921
Metalloproteinase inhibitor 1	994.74	9.9582	0.00289	2.5387
Coronin-1A	0.41347	-1.2742	0.00371	2.4303
Ig kappa chain V-II region FR	0.25858	-1.9513	0.00380	2.4202
6-phosphogluconate dehydrogenase, decarboxylating	0.30418	-1.717	0.00584	2.2332
Cytosolic non-specific dipeptidase	0.21682	-2.2054	0.00693	2.1591
Myc target protein 1	283.31	8.1462	0.00878	2.0564
Target of Nesh-SH3	1769.6	10.789	0.00894	2.0489
Complement C1q subcomponent subunit B	0.4726	-1.0813	0.00950	2.0224
Thymidine phosphorylase	0.28019	-1.8355	0.00975	2.0109
Guanine nucleotide-binding protein Gz subunit alpha	0.36684	-1.4468	0.01027	1.9883
Protein S100-A6	0.31982	-1.6447	0.01159	1.936
Calpain small subunit 1	6.7488	2.7546	0.01422	1.8471
Dynactin subunit 2	548.29	9.0988	0.01442	1.841
Tyrosine-protein phosphatase non-receptor type 6	0.25594	-1.9661	0.01491	1.8266
Ig kappa chain V-I region Lay	267.29	8.0623	0.01525	1.8166
Guanine nucleotide-binding protein GI/GS/GT subunit beta-1	0.472	-1.0831	0.01563	1.8061
Mucin-1	862.22	9.7519	0.01930	1.7144
Fatty acid synthase	0.088116	-3.5045	0.02117	1.6743
Latent-transforming growth factor beta-binding protein 1	8.6911	3.1195	0.02123	1.673
Glucose-6-phosphate isomerase	0.1888	-2.4051	0.02156	1.6664
Kinesin-like protein KIF2A	0.30112	-1.7316	0.02222	1.6533
Superoxide dismutase Cu-Zn	0.30435	-1.7162	0.02225	1.6527
Ig kappa chain V-I region Wes	3.0285	1.5986	0.02655	1.576
Rap1 GTPase-activating protein 2	0.2397	-2.0607	0.02774	1.5569
Major vault protein	806.86	9.6562	0.02864	1.5431
Junction plakoglobin	5.1189	2.3558	0.03180	1.4976
Serine/threonine-protein phosphatase PP1-alpha catalytic subunit	0.31304	-1.6756	0.03312	1.4799
Desmoplakin	5.7687	2.5282	0.03433	1.4644
Heat shock 70 kDa protein 1A	2.8601	1.516	0.03515	1.4541
Caldesmon	0.31793	-1.6532	0.03573	1.4469
Ig kappa chain V-I region Ka	3.3146	1.7288	0.03695	1.4324
Ig alpha-2 chain C region	2.6341	1.3973	0.03806	1.4195
Ficolin-1	5.762	2.5266	0.03842	1.4154
Thrombospondin-4	2.6685	1.416	0.03846	1.415
Protein S100-A8	287.2	8.1659	0.04828	1.3163

Proteins commonly identified in breast cancer EVs[1]

MDA-MB231 and YWBC EVs	MCF10DCIS.com and YWBC EVs
Serpin B3	Mucin-1
Tripeptidyl-peptidase 2	Protein S100-A8
Prolactin-inducible protein	Galectin-7
Clatherin light chain A	Serpin B3
Tetraspanin-15	Prolactin-inducible protein
Vasorin	Clatherin light chain A
Proteasome subunit beta type-4	CD109 antigen
Proteasome subunit alpha type-1	Peflin
Programmed cell death 6-interacting protein	Focal Adhesion Kinase 1
	Programmed cell death 6-interacting protein

[1]The unique proteins identified in YWBC patients but not healthy donors were compared to the proteins identified in breast cell lines, as shown in the Venn diagram in Figure 3a (9 overlapping proteins with MDA-MB231).
